# Investigation Into Different Wood Formation Mechanisms Between Angiosperm and Gymnosperm Tree Species at the Transcriptional and Post-transcriptional Level

**DOI:** 10.3389/fpls.2021.698602

**Published:** 2021-07-02

**Authors:** Hui Li, Guanghui Chen, Hongying Pang, Qiao Wang, Xinren Dai

**Affiliations:** ^1^State Key Laboratory of Tree Genetics and Breeding, Chinese Academy of Forestry, Beijing, China; ^2^Guangzhou Institute of Forestry and Landscape Architecture, Guangzhou, China; ^3^Shandong Peanut Research Institute, Shandong Academy of Agricultural Sciences, Qingdao, China; ^4^Research Institute of Forestry, Chinese Academy of Forestry, Beijing, China

**Keywords:** angiosperm, gymnosperm, transcriptome, co-expression network, alternative splicing, wood formation

## Abstract

Enormous distinctions of the stem structure and cell types between gymnosperms and angiosperms tree species are expected to cause quite different wood physical and mechanical attributes, however, the molecular mechanisms underlying the differing wood morphology are still unclear. In this study, we compared the transcriptomes obtained by RNA-Seq between *Populus alba* × *P. glandulosa* clone 84K, and *Larix kaempferi* (Lamb.) Carr trees. Available genome resource served as reference for *P. alba* × *P. glandulosa* and the Iso-Seq results of a three-tissues mixture (xylem, phloem, and leaf) were used as the reference for *L. kaempferi* to compare the xylem-specifically expressed genes and their alternative splicing model. Through screening, we obtained 13,907 xylem-specifically expressed genes (5,954 up-regulated, 7,953 down-regulated) in the xylem of *P. alba* × *P. glandulosa*, and 2,596 xylem-specifically expressed genes (1,648 up-regulated, 948 down-regulated) in the xylem of *L. kaempferi*. From the GO and KEGG analyses, some genes associated with two wood formation-related pathways, namely those for phenylpropanoid biosynthesis, and starch and sucrose metabolism, were successfully screened. Then the distributions and gene expression models between *P. alba* × *P. glandulosa* and *L. kaempferi* in those pathways were compared, which suggested differential wood formation processes between the angiosperm and gymnosperm trees. Furthermore, a Weight Gene Co-expression Network Analysis (WGCNA) for total xylem-specifically expressed genes in two species was conducted, from which wood formation-related modules were selected to build a co-expression network for the two tree species. The genes within this co-expression network showed different co-expression relationships between the angiosperm and gymnosperm woody species. Comparing the alternative splicing events for wood formation-related genes suggests a different post-transcriptional regulation process exists between the angiosperm and gymnosperm trees. Our research thus provides the foundation for the in-depth investigation of different wood formation mechanisms of angiosperm and gymnosperm species.

## Introduction

Seed-bearing plants are the primary plant species on our planet and are composed of two main phyla, gymnosperms and angiosperms, which appeared ca. 300 million years ago (Pavy et al., 2012). Gymnosperms include seed-bearing plants that are woody, herbaceous, or climbing (vines), consisting of four extant sub-phyla: Cycadophyta (cycads), Ginkgophyta (*Ginkgo biloba* L.), Coniferophyta (conifers), and Gnetophyta (gnetophytes). Among them, the conifers are the most numerous gymnosperms found on earth, comprising 50 genera and 550 species, and they are widely distributed throughout the Northern Hemisphere (Carvalho et al., 2013). For the angiosperms, taxonomists have identified about 352,000 species, making it the most diversiform group on earth (www.theplantlist.org). All of these angiosperm species originated from one single ancestor ca. 167–199 million years ago (Bell et al., 2010), and diverged into eight extant clades, namely the Amborellales, Nymphaeales, Austrobaileyales, Monocots, Magnoliids, Ceratophyllales, Chloranthales and Eudicots. Among them, the Eudicots is the primary clade, containing about 262,000 species (Zeng et al., 2014).

Wood represents a renewable natural resource for feedstocks used in several bio-economy products, such as pulp, paper, and biomaterials, and potentially biofuels as well. It is also a major carbon sink in natural ecosystems. The stem of woody plants is mainly composed of secondary xylem (Li et al., 2010). The most striking divergent characteristics between gymnosperm and angiosperm wood are in their anatomical structure and chemical composition (Jokipii-Lukkari et al., 2018). In gymnosperm trees, tracheids provide both water transport and mechanical support; in angiosperms, vessel elements are responsible for carrying water, with fibers providing mechanical support for the stem. To some extent, however, the tracheids in gymnosperms are similar to the vessel in angiosperms (Courtois-Moreau et al., 2009; Dieset, 2011). Vessel elements appear only in angiosperm species and are shaped by the need for rapid and efficient water transport capacity (Sperry et al., 2006). Wood formation in tree species requires the complex coordination of two highly ordered processes, cell differentiation and secondary cell wall (SCW) thickening, which are initiated from the vascular cambium and result in thick-walled xylem cells (Zhao et al., 2014). Secondary walls of xylem cells are composed of high-content cellulose and lignin (Li et al., 2010). Importantly, the amount and chemical structure of lignin in SCWs differs between gymnosperms and angiosperms, and these differences are closely related to plant evolution (Vanholme et al., 2010; Nawawi et al., 2016). The lignin in gymnosperms is exclusively polymerized from guaiacyl (G) units, while both G units and syringyl (S) units are the major components of lignin in angiosperms (Sarkanen, 1971; Higuchi et al., 1977; Boerjan et al., 2003; Weng et al., 2008).

In the last few decades, significant progress has been made in uncovering the molecular players involved in SCW biosynthesis in tree species, including hormonal signals, receptor kinases, and the transcriptional network which controls SCW formation. Both NAC and MYB master switches and their downstream transcription factors (TFs) have been shown to play critical roles during SCW formation (Yamamoto et al., 1997; Aspeborg et al., 2005; Li et al., 2012; Zhong and Ye, 2014; Ye and Zhong, 2015; Zhang et al., 2018; Du et al., 2019; Wang et al., 2019). Genes encoding the enzymes for the biosynthesis of SCW cellulose, hemicelluloses, and lignin have been identified and most of them are now functionally characterized in *Populus* and *Eucalyptus* (Suzuki et al., 2006; Shi et al., 2009; Lu et al., 2013; Yuan et al., 2014; Ye and Zhong, 2015; Kim et al., 2019; Wang et al., 2019). Many recent studies aiming to identify those key genes involved in wood development in many plant species were carried out to investigate xylem evolution at the transcriptome level (Cronk and Forest, 2017; He and Groover, 2017; Sundell et al., 2017; Tuskan et al., 2018; Roodt et al., 2019). The expanding genomic resources of tree species are invaluable for exploring secondary xylem formation and its evolution. Yet woody gymnosperm species typically have large genome sizes and high heterozygosity, so their transcriptome analyses remain limited by the poor quality of assembled genomes.

Alternative splicing (AS) is an important model of post-transcriptional regulation that can increase transcriptional and proteomic diversity in eukaryotic organisms (Chen and Manley, 2009). AS that combines different transcript splice junctions results in transcripts with shuffled exons, alternative 5′ or 3′ splicing sites, retained introns, and different transcript termini. In plants, 33−60% of the mRNAs are alternatively spliced (Shen et al., 2014), of which more than 60% are in the form of retained introns (Zhang et al., 2010; Syed et al., 2012). Transcriptome analysis has revealed that approximately 36% of wood-expressed genes undergo AS in the xylem of *P. trichocarpa* (Bao et al., 2013), and that 28.3% and 20.7% of the highly expressed transcripts in developing xylem tissue undergo AS events in *Populus* and *Eucalyptus*, respectively (Xu et al., 2014). Interestingly, most of the key TFs in the first layer of SCW regulatory network also undergo AS. In *Populus, PtrSND1-A2*^*IR*^, a splice variant of stem-differentiating xylem (SDX)- SND1, acts as a dominant negative regulator of the SND1 transcriptional network (Li et al., 2012). *PtrSND1-A2*^*IR*^ is derived from *PtrSND1-A2* (also named *PtrWND1B/PtrVNS11*), having lost its DNA binding and activation domain but retaining its dimerization capability; it represses the transcription of *PtrSND1* members and their target gene *PtrMYB021* by translocating into the nucleus exclusively as a heterodimeric partner with full-size PtrSND1s (Li et al., 2012). This is the first time to report about the TF family's auto-repression by its own splice variant found in plants (Zhang et al., 2018; Camargo et al., 2019). Other research has shown that overexpression of *PtrSND1-A2* enhances fiber cell wall thickening, while overexpression of *PtrSND1-A2*^*IR*^ (*PtrWND1B-l*) inhibits the fiber cell wall-thickening process (Zhao et al., 2014). *VND6*, another key TF in the first layer of the SCW regulatory network, was also confirmed to undergo alternative splicing during wood formation in poplar (Lin et al., 2017). Its splice variant, *PtrVND6-C1*^*IR*^ derived from *PtrVND6-C1* (*PtVNS01/ PtrWND5A*), suppresses the protein functioning of all *PtrVND6* and *PtrSND1* family members, whereas *PtrVND6-C1*^*IR*^ is unable to suppress *PtrSND1-A2*; further, *PtrSND1-A2*^*IR*^ has no effect on *PtrVND6-C1*^*IR*^. Both *PtrVND6-C1*^*IR*^ and *PtrSND1-A2*^*IR*^ function together in the reciprocal cross-regulation of VND and SND members to maintain homeostasis during xylem differentiation and plant development. Recently, for a gymnosperm species, *LaSCL6*, a member of the GRAS transcription factor family, was determined to have two variants which are differentially expressed during the growth and development in *L. kaempferi* (Zang et al., 2019). Our knowledge of AS of other genes, especially the key TFs in SCW regulatory network, is still vastly limited.

Although some molecular players are distinguished by their conserved functions during wood formation in gymnosperms and angiosperms, wood formation between gymnosperms and angiosperms is clearly different (i.e., xylem cell type and wood composition, among others). This suggests their gene regulatory networks are also not the same and that certain genes may have distinct functions in the two tree species above. Here, we employed next-generation sequencing and Iso-Seq technology to explore the gene expression model across wood-forming tissues in the angiosperm *P. alba* × *P. glandulosa* and the gymnosperm *L. kaempferi*. By screening xylem specific-expressed genes, associating these genes with wood-formation related pathways, building a co-expression network and analyzing the AS of common genes among the two tree species, differential mechanisms of wood formation between angiosperm and gymnosperm tree species were investigated and preliminarily discussed. Our research provides theoretical support for searching for the differing wood formation mechanisms between angiosperm and gymnosperm species.

## Materials and Methods

### Plant Materials Collection and RNA Extraction

Samples were collected from 10-year-old *P. alba* × *P. glandulosa* trees in the Beiwu garden (39°59′N, 116°15′E; Beijing, China) and 10-year-old *L. kaempferi* tress in the Dagujia seed orchard (42°22′N, 124°51′E; Liaoning Province, China) as describe in He et al. (2020). Briefly, the bark and phloem were peeled off at breast height, the immature xylem and phloem were collected from each species by scratching with single-end razors. These, along with leaf samples, were immediately frozen in liquid nitrogen. Each sample was collected from three trees per species as three biological replicates. Total RNAs were extracted by using the improved CTAB method (Lorenz et al., 2010). The RNA was precipitated by ethanol, and then dissolved in RNase-free water. After digesting it with DNaseI, we used the Agilent 2100 Bioanalyzer and NanoDrop to measure the RNA concentration, RIN value, 28s/18s value, and size of the fragment, to confirm the integrity and purity of RNA in the samples.

### Construction of a PacBio Library With Different Library Sizes and Sequencing

To correct the Hi-Seq results and analyze AS events in the two tree species, we also constructed PacBio libraries. By taking 1 μg RNA subsamples, first-strand cDNA was synthesized using the Clontech SMARTer PCR cDNA synthesis Kit (cat. no. 634926, http://www.clontech.com/), with an anchored olig (dT) 30 as the primer. Then the double-strand cDNA was amplified by carrying out a long PCR (LD-PCT) using the Advantage 2 PCT kit (Clonetech, cat. No.639206). The 1–2 kb, 2–3 kb, and 3–6 kb cDNA fragments were generated by the BluePinppin size-selection system (Sage Science, http://www.sagescience.com/). For those transcripts whose size was more than 3 kb, we generated the BluePippin selection again. The PacBio libraries were then constructed with a Pacific Biosciences SMARTbell template Prep Kit 1.0 (part 100-259-100, http://www.pacb.com/), following the manufacture's protocol. These libraries were later sequenced on a PacBio RSII real-time (RT) platform, using the SMRT Cell 8 Pac v3 (part100-171-800) with a total of 8 SMRT cells: that is, the 1–2 k and 2–3 k libraries were each sequenced with 3 SMRT cells, while the 3–6 k libraries were sequenced with 2 SMRT cells.

### RNA-Seq Library Construction and Sequencing

We used magnetic beads with Oli (dT) to enrich the mRNA and broke it into pieces by applying a fragmentation buffer. Employing these mRNAs as the template, random hexamers were used to synthesize the first-strand cDNA. Next, together with buffer, the dNTPs, RNaseH, and DNA polymerase I synthesized the second-strand cDNA. Each cDNA library was purified and to it a joint end with A was added. Following their PCR amplification, the ensuing PCR products were sequenced by the Illumina HiSeq 2500 platform to generate 100-bp paired sequence reads.

### Analysis of PacBio Single-Molecule Reads

We used the SMRT Analysis Server (v2.3) to handle the Raw SMRT sequencing reads in order to obtain the Full-Length non-chimeric Read (FLNC). The first step in doing this was to properly deal with the inserted anti-molecular sequence and assess the length of cDNAs loaded into SMRT cell using these parameters “minFullPasses” = 0, “minPredictedAccruacy” = 80, “numThreads” = 12. Reads-of-Insert can be obtained from a single molecular inserted sequence. After removing the cDNA primer and polyA, we classified the Reads-of-Insert into full-length or non-full-length, chimeric or non-chimeric, fragments with these two parameter settings: “min_seq_len” = 300, “cpus” = 12. The full-length and non-chimeric transcripts were corrected, using the Interactive Clustering and Erro Correction (ICE) algorithm, to obtain the corresponding sequences. Then the cover rating for these corresponding sequences was predicted, after which the non-full length and non-chimeric sequences were corrected by the Quiver algorithm.

Completing these steps left us with high- and low-quality full-length transcripts. The LSC software was then used to correct the FLNC by referencing short reads of HiSeq (Au et al., 2012). For each corrected FLNC, we used the CD-HIT-EST to reduce the redundant highly similar transcripts (Li and Godzik, 2006); these transcripts were merged by Cogent software to obtain the unigenes. For their annotation, unigenes were BLAST-searched to NR (ftp://ftp.ncbi.nlm.nih.gov/blast/db), NT (ftp://ftp.ncbi.nlm.nih.gov/blast/db), COG (http://www.ncbi.nlm.nih.gov/COG), KEGG (http://www.genome.jp/kegg) and Swiss-Prot (http://ftp.ebi.ac.uk/pub/databases/swissprot) databases. According to those annotations of the NR database, Blast2GO (https://www.blast2go.com) (Conesa et al., 2005) was used to obtain the annotations from the GO database. According to the latter, the CDs of best-matched unigenes were selected for further analysis. For those unigenes that could not be annotated by any database, ESTscan software was relied upon to build a model and predict the CDs for them (Iseli et al., 1999).

### Read Mapping and Differentially Expressed Genes (DEGs) Analyses

Raw data were filtered using the NGS QC Toolkit to obtain clean reads. These were mapped onto the genome or PacBio Isoform transcripts via Hierarchical Indexing for Spliced Alignment of Transcripts (HISAT) (Kim et al., 2015). The mapped output was processed by Cufflinks to obtain the Fragments per Kilobase Million (FPKM) for all genes in each sample, for which correlations among different replicate samples were determined by calculating Pearson correlation. Using the DEseq2 software (Love et al., 2014), we obtained the DEGs among the types of plant tissue. The DEGs in the two species were filtered according to these criteria: the DEGs that had a log_2_ (X vs. P or L) ≥ 1 (Q-value ≤ 0.05) were designated as up-regulated genes in xylem compared to phloem and leaf parts; those DEGs that met log_2_ (X vs. P or L) ≤ −1 (*Q*-value ≤ 0.05) were considered down-regulated genes compared with the other two tissue types. The intersection of X vs. P and X vs. L correspond to the xylem-specifically expressed genes.

### GO and KEGG Analysis for Xylem-Specifically Expressed Genes

For all DEGs, their Gene Ontology (GO) enrichment analysis was conducted using GOseq and topGO (Young et al., 2010). To do this, we first mapped all the detected genes in the two tree species to the GO database (http://geneontology.org/), to obtain their GO annotations. According to these results, we enriched the xylem-specifically expressed genes by comparing them to the reference gene background by applying the hypergeometric test. The gene numbers for each term were then calculated, among which the significantly enriched GO terms were determined. We also mapped all detected genes to the Kyoto Encyclopedia of Genes and Genomes (KEGG) database (https://www.kegg.jp/), to obtain their corresponding KEGG annotations. According to the KEGG annotations, we enriched the xylem-specifically expressed genes in the main biochemical and signal transduction pathways and identified those significantly enriched metabolic pathways or signal transduction pathways. The “phyper” package for the R computing platform was used to calculate the *p* values, as described in Li et al. (2020).

### Association of mRNAs in Wood Formation-Related Pathways

Based on the KEGG results, we located the enriched xylem-specifically expressed genes in two pathways, the phenylpropanoid biosynthesis pathway, and the starch and sucrose pathway, to derive and display a putative gene expression model. These pathways were visualized in Chewdraw software (version Professional 15.0). The heatmaps were drawn with TBtools 0.6669 (Chen et al., 2020).

### Weighted Gene Co-expression Network Analysis (WGCNA) for Xylem-Specifically Expressed Genes

We used “WGNCA” package in R software to carry out a co-expression network analysis for xylem-specifically expressed genes in the two tree species. Then, for each module per species, we also conducted GO and KEGG analyses. After combining these GO and KEGG results, we selected the modules in which wood formation-related genes were evidently enriched.

### Network Building for Wood-Formation Related Genes

To discern the possible relationships among wood formation-related genes, we chose the top 50,000 gene relation pairs in the selected edge file of WGCNA and imported these pairs into Cytoscape software (Kohl et al., 2011). According to the GO and KEGG analyses and BLASTn results in the selected module of each species, we built co-expression networks of wood formation-related genes in Cytoscape software to depict the co-expression relationships of the two tree species.

### Identification of Alternative Splicing (AS)

To classify the AS events, Cogent software was used to reconstruct the PacBio transcripts and this yielded the UniTransModels. After blasting the non-redundant transcripts to UniTransModels, using GMAP software, the output results from GMAP were inputted into SUPPA software to detect the AS events (Li et al., 2017).

### Verification of RNA-Seq Result and AS Events

To validate the RNA-Seq and AS events experimentally, PCRs were run for eight pairs of homologous genes that underwent different AS types in the two tree species. For each sample, 1 mg of total RNA was reverse-transcribed into first-strand cDNA, by using the PrimeScript RT reagent Kit gDNA Eraser (Takara, Dalian, China). The primers were designed based on the consensus gene sequences of the two tree species, and these primer sequences are listed in Supplementary Tables 1A,B. For validation of RNA-Seq results, we chose several genes from both *P. alba* × *P. glandulosa* and *L. kaempferi* to perform the qRT-PCR, by following the instructions for SYBR® Premix Ex Taq™ II (Takara), for the xylem, phloem, and leaf tissues, respectively. Linear fitting between RNA-Seq and qRT-PCR data was done in Origin 2016 software. To validate the AS events, the PCR was run in a 25-ml reaction system, using the High-Fidelity PCR Master Mix (NEB), whose procedure went as follows: initial denaturation at 98°C for 1 min, 98°C for 10 s, 60°C for 30 s, and 72°C for 1–3 min (35 cycles) and a final extension at 72°C for 5 min. All the PCR products were visualized by 1.5% agarose gel electrophoresis analysis.

## Results

### Global Analysis of RNA-Seq for the Two Tree Species

**Figure 1:**
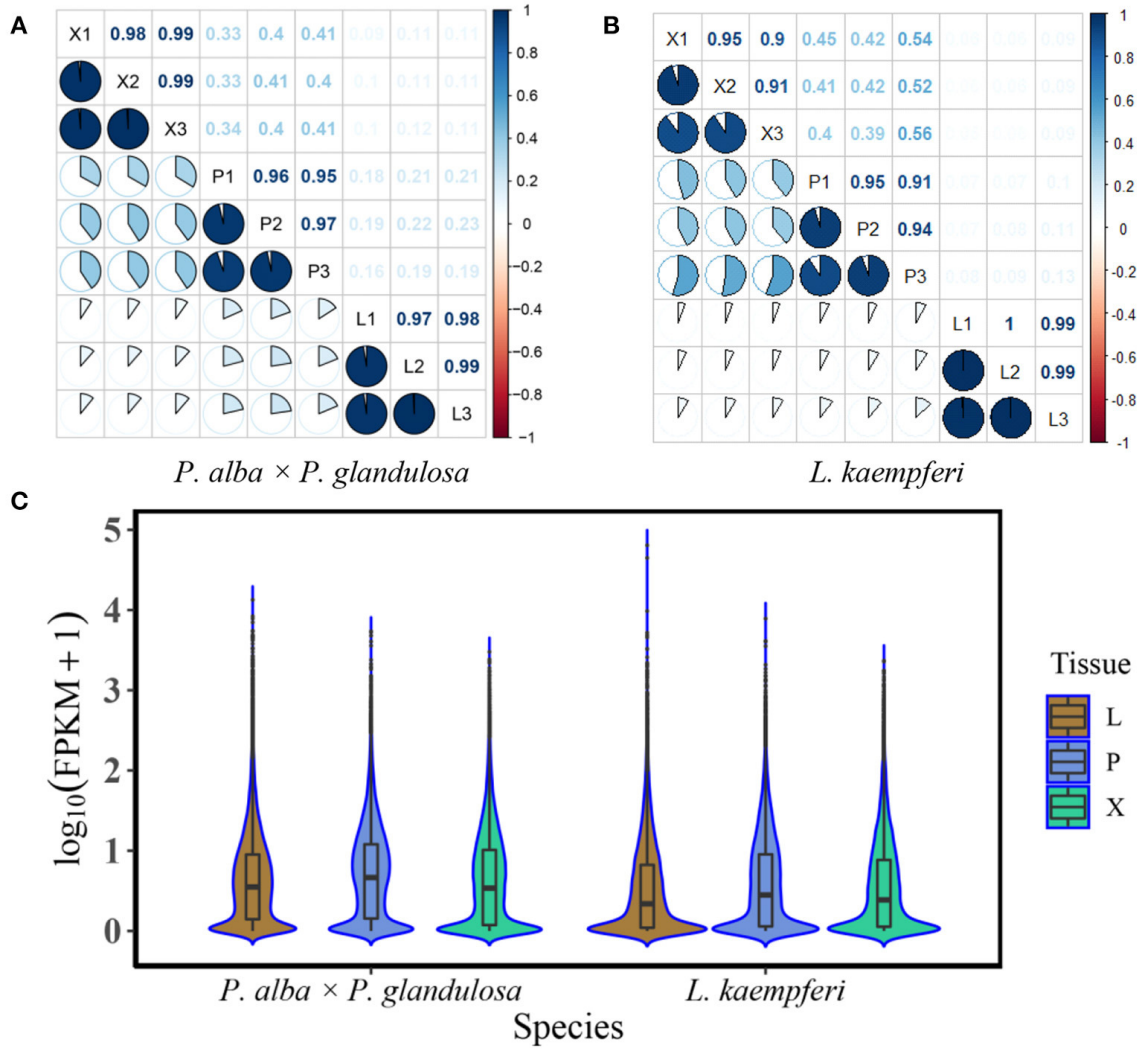
Correlation analysis and the gene expression distributions of different tissues in the two tree species. **(A)** Correlations among different tissues and replicates of the angiosperm *Populus alba* × *P. glandulosa* clone 84K (*P. alba* × *P. glandulosa*). **(B)** Correlations among different tissues and replicates of the gymnosperm *Larix kaempferi* (Lamb.) Carr (*L. kaempferi*). **(C)** Gene expression distribution of different tissues (Xylem: X; Phloem: P; Leaf: L) in two tree species. Box plots appear in the center of the violin graphs. The top edge, middle edge, and bottom edge represent third quartile, median, and first quartile, respectively. The expression levels of the genes were standardized by the log_10_ (FPKM) algorithm. FPKM represents Fragments per Kilobase Million.

We conducted the RNA-Seq of xylem, phloem, and leaf tissues of two tree species, with three biological replicates per tissue. Before the formal data analysis, we tested for correlations (using Pearson's r) among the different replicates and tissue types (Figure 1). The tests confirmed that the three replicates of the same tissue in each of the species were strongly correlated, having *r* values > 0.8 (Figures 1A,B), thus showing that their transcriptome data was suitable for further analysis. We also derived key statistics for gene expression levels in different tissue of the two tree species. In*P. alba* × *P. glandulosa*, two high proportions of expressed genes focus at 0.1 and 1.0, respectively. By contrast, there was only one high proportion of expressed genes focus at 0.1 in *L. kaempferi*. The median gene expression levels in the three tissues of *P. alba* × *P. glandulosa* were about 0.8–0.9, and higher than those of *L. kaempferi* (Figure 1C). These phenomena reflected different gene expression distributions between angiosperm and gymnosperm tree species.

We next profiled the RNA transcriptome for three tissues (xylem, phloem and leaves) of the two tree species. In *P. alba* × *P. glandulosa*, we obtained 50,995,164, 56,113,145, and 50,995,164 clean reads in the xylem, phloem, and leaves, respectively; in *L. kaempferi*, the corresponding counts were 66,463,409, 77,166,823, and 63,449,442 clean reads. The reads from *P. alba* × *P. glandulosa* were mapped to the reference genome (https://bigd.big.ac.cn/), while those reads from *L. kaempferi* were mapped to the SMRT library which had corrected by RNA-Seq data. The mapping rates for xylem, phloem, and leaf tissue were 95.39 95.41 and 95.60 for *P. alba* × *P. glandulosa*, and 46.79 41.41 and 54.95 for *L. kaempferi*, respectively. Eventually, we obtained 44,561 genes in xylem, 47,465 in phloem, and 48,091 in leaves of *P. alba* × *P. glandulosa*, and likewise, 21,535 genes in xylem, 22,986 in phloem and 23,233 in leaves of *L. kaempferi* (Table 1).

**Table 1:** Summary information of the RNA-Seq results for the two tree species.

**Species**	**Tissue**	**Total reads**	**Mapped reads**	**Mapping rate%**	**Number of gene**
*Populus alba × P. glandulosa*	xylem	50,995,164	48,644,287	95.39	44,561
	phloem	56,113,145	53,537,552	95.41	47,465
	leaf	50,995,164	48,751,377	95.60	48,091
*Larix kaempferi*	xylem	66,463,409	24,451,888	46.79	21,535
	phloem	77,166,823	31,954,781	41.41	22,986
	leaf	63,449,442	34,865,468	54.95	23,233

### Screening of Xylem-Specifically Expressed Genes in the Two Tree Species

**Figure 2:**
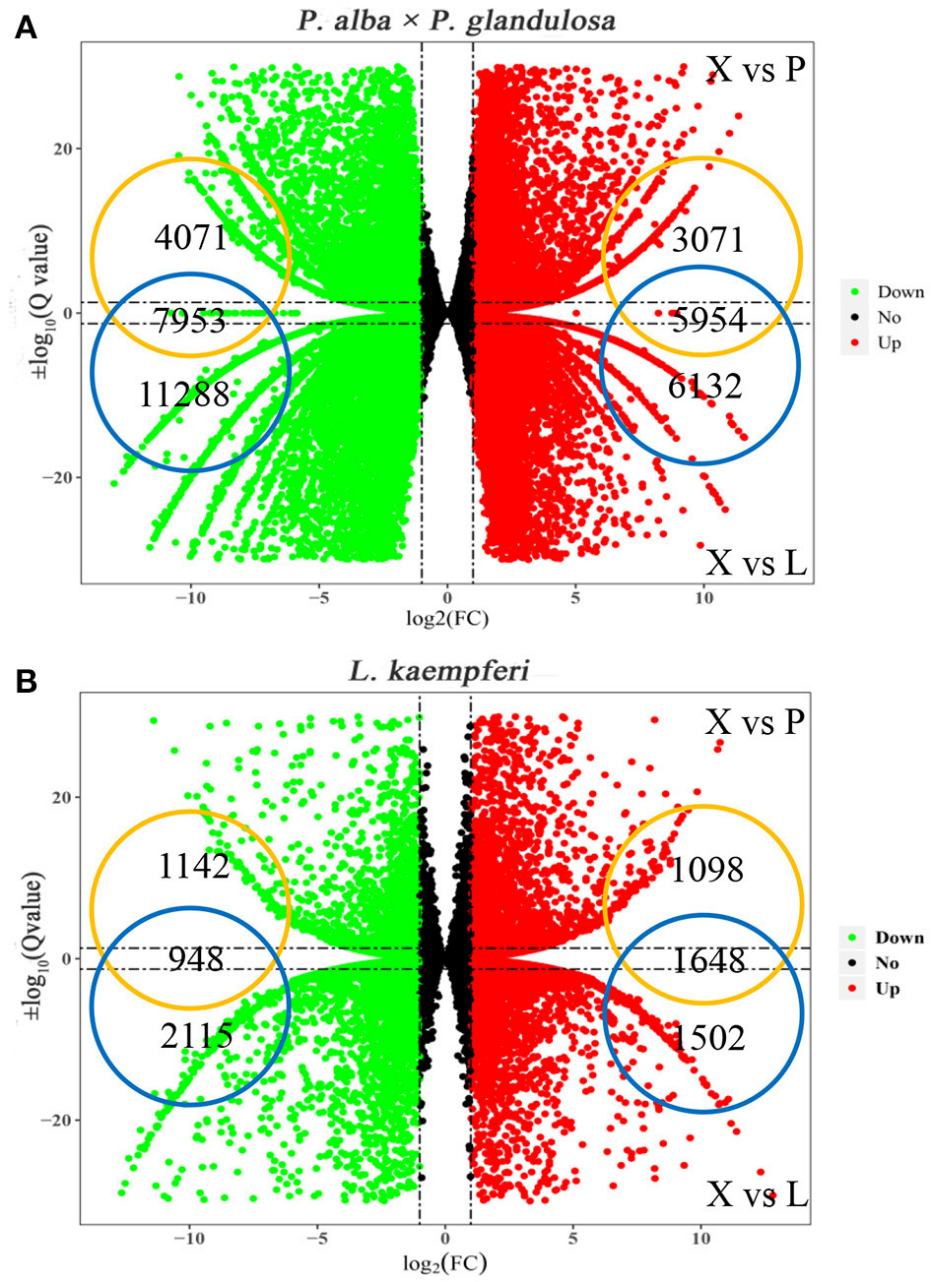
Distributions of up- and down-regulated DEGs in the two tree species. **(A)** Up and down-regulated DEGs in *P. alba* × *P. glandulosa*. **(B)** Up and down-regulated DEGs in *L. kaempferi*. In both panels, X vs. P represents the differential expression analysis between the xylem and phloem; likewise, X vs. L represents the differential expressed analysis between the xylem and leaf. The vertical dotted lines correspond to the log_2_ (fold- change) = ±1, while the horizontal dotted lines are positioned at log_10_ (*Q*-value) = ±1.3 (*Q*-value = 0.05). The genes at the intersection of two groups are the up- and down-regulated genes in xylem tissue.

To understand the expression discrepancy of RNAs in three different tissues of the two tree species, we conducted a DEG analysis for *P. alba* × *P. glandulosa*, we obtained 9,025 up-regulated DEGs and 12,024 down-regulated DEGs in the X vs. P group, and 12,086 up-regulated DEGs and 19,241 down-regulated DEGs in the X vs. L group (Figure 2A; Supplementary Tables 2A,B). For *L. kaempferi*, we obtained 2,746 up-regulated DEGs and 2,080 down-regulated DEGs in the X vs. P group, and 3,150 up-regulated DEGs and 3,063 down-regulated DEGs in the X vs. L group (Figure 2B; Supplementary Tables 2C,D). By taking the overlapping intersection of X vs. P and X vs. L, finally, we finally obtained 5,954 up-regulated DEGs and 7,953 down-regulated DEGs in the xylem of *P. alba* × *P. glandulosa* (Figure 2A), and 1,648 up-regulated and 948 down-regulated DEGs in the xylem of *L. kaempferi* (Figure 2B).

### Validation of RNA-Seq Results by qRT-PCR

**Figure 3:**
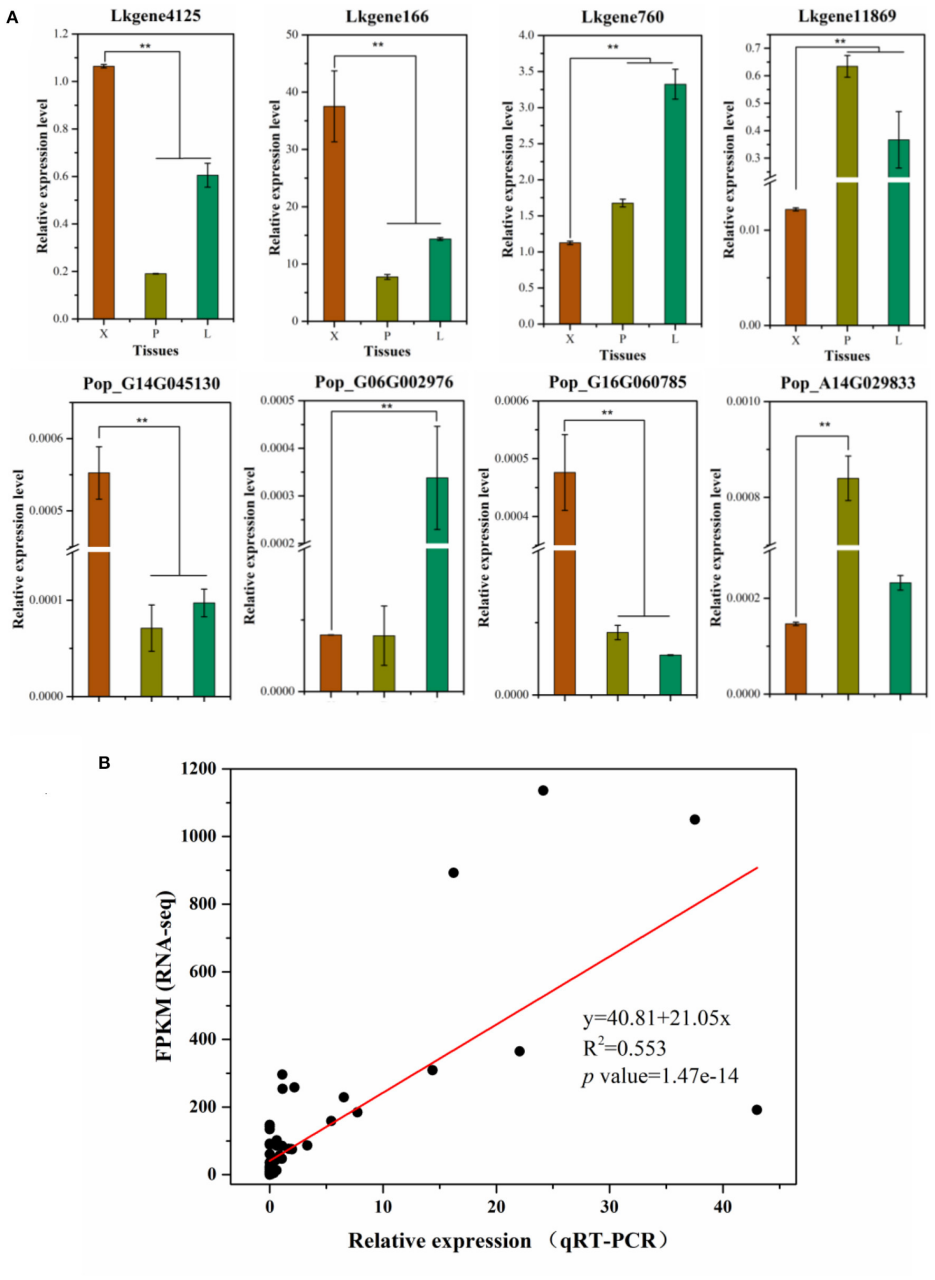
Validation of RNA-Seq results by qRT-PCR. **(A)** The qRT-PCR results for selected genes in the two tree species. **(B)** Linear regression for the RNA-Seq and qRT-PCR results. In this graph, gene expression levels in RNA-Seq are standardized by FPKM, the expression levels in qRT-PCR were standardized by the 2^−Δ*ct*^ algorithm. In **(A)**, the relative expression levels in the three tissue types are shown as the mean ± sd; the ** indicates a significant difference at *P* < 0.01 according to a one-way ANOVA.

To detect and check the robustness of our RNA-Seq results, we selected four genes in *P. alba* × *P. glandulosa*: Pop_G14G045130 (*PagBXL2)*, Pop_G16G060785 (*PagIRX9*), Pop_G06G002976 (*PagPME2*), and Pop_A14G029833 (*PagARA12*), and similarly four genes in *L. kaempferi*: Lkgene4125 (*LkIRX9H*), Lkgene760 (*LkMSR2*), Lkgene166 (*LkC4H*), and Lkgene11869 (*LkSUS4*). These were used to perform qRT-PCR in xylem, phloem, and leaf tissues (Figure 3). In *P. alba* × *P. glandulosa*, except for Pop_G06G002976, the other three genes were significantly up-regulated or down-regulated in xylem when compared to the other tissues. In *L. kaempferi*, all four selected genes were significantly up-regulated or down-regulated in xylem (Figure 3). Hence, the qRT-PCR results agreed with the RNA-Seq analysis. The linear relationship between the RNA-Seq and qRT-PCR results was fitted in the Origin software; this showed their data were significantly correlated (*p* value = 1.47E-14). The slope of the fitted line was 20.15, indicating a positive relationship between RNA-Seq and qRT-PCR results, and the goodness-of-fit was *R*^2^ = 0.553 (Figure 3). Taken together, these results indicated our RNA-Seq results were reliable and useful for further analysis.

### GO Analysis and Functional Classification for Xylem-Specifically Expressed Genes

By filtering the DEGs, we obtained 13,907 xylem-specifically expressed genes (5,954 up-regulated, 7,953 down-regulated) in *P. alba* × *P. glandulosa*, and 2,596 xylem-specifically expressed genes (1,648 up-regulated, 948 down-regulated) in *L. kaempferi*. To assess and compare the functional differences of these genes between the two species, we implemented GO and KEGG analyses. Three main ontologies—biological process (BP), cellular component (CC), and molecular function (MF)—were considered here. To fully display the GO terms, we reordered them according to the *Q*-values of the GO results and selected only the top 10 GO terms in each ontology category for the visualization.

In *P. alba* × *P. glandulosa*, 13,907 xylem-specifically expressed genes were clustered in 50 GO terms. Under BP, genes were significantly enriched in single organism process (GO: 0044699, *Q*-value = 1.4E-07), localization (GO: 0051179, *Q*-value = 5.45E-05), and growth (GO: 0040007, *Q*-value = 0.017); under MF, genes were significantly enriched in transporter activity (GO: 0005215, *Q*-value = 2.20E-14) and electron carrier activity (GO: 0009055, *Q*-value = 1.1E-4); finally, under CC, genes were significantly enriched in membrane (GO: 0016020, *Q*-value = 3.16E-58), membrane part (GO: 0044425, *Q*-value = 3.16E-58), supramolecular fiber (GO: 0099512, *Q*-value = 1E-5), extracellular region (GO: 0005576, *Q*-value = 1.1E-3), cell junction (GO: 0030054, *Q*-value = 2.37E-2), and symplast (GO: 0055044, *Q*-value = 2.37E-2) (Supplementary Table 3A). In *L. kaempferi*, 2,596 xylem-specifically expressed genes were enriched in 43 GO terms. Under BP, genes were significantly enriched only in single organism process (GO: 0044699, *Q*-value = 6.84E-09); under MF, genes were significantly enriched in catalytic activity (GO: 0005576, *Q*-value = 1.903E-33), nucleic acid binding transcription factor activity (GO: 0099512, *Q*-value = 6.0E-08), and structural molecule activity (GO: 0044425, *Q*-value = 9.05E-3); under CC, genes were significantly enriched in membrane part (GO: 0003824, *Q*-value = 3.486E-11), membrane (GO: 0001071, *Q*-value = 2.39E-2), and nucleoid (GO: 0005198, *Q*-value = 2.7E-2) (Supplementary Table 3C). In Supplementary Figures 1A,C, more detailed GO terms are shown for the two species. According to the GO results, genes in *P. alba* × *P. glandulosa* tend to increase plant growth and enable the directed movement of substances within cells; in contrast, genes in *L. kaempferi* involved in the structural integrity of a complex or its assembly within or outside a cell, catalyzed reactions process. In sum, our GO analysis results indicated different functions between the angiosperm and gymnosperm trees.

For the KEGG analysis, we reordered the results according to the Rich factor and chose the top 30 pathways for visualization purposes. In *P. alba* × *P. glandulosa*, 13,907 xylem-specifically expressed genes were enriched in 132 pathways. The top three significantly enriched pathways were photosynthesis (ko00195), metabolic pathways (ko01100), and photosynthesis-antenna proteins (ko00196) (Supplementary Figure 1B; Supplementary Table 3B) with corresponding enriched gene numbers of 99, 1,605, and 34 respectively. In *L. kaempferi*, 2,596 xylem-specifically expressed genes were clustered in 112 pathways; its top three significantly enriched pathways were starch and sucrose metabolism (ko00500), flavonoid biosynthesis (ko00941), and metabolic pathways (ko01100). The enriched gene numbers respectively were 196, 59, and 655 (Supplementary Figure 1D; Supplementary Table 3D). Hence, these KEGG results also uncovered different gene functions during the metabolism process between the two tree species.

### Association of Xylem-Specifically Expressed Genes With Lignin and Cellulose-Formation Related Pathways

**Figure 4:**
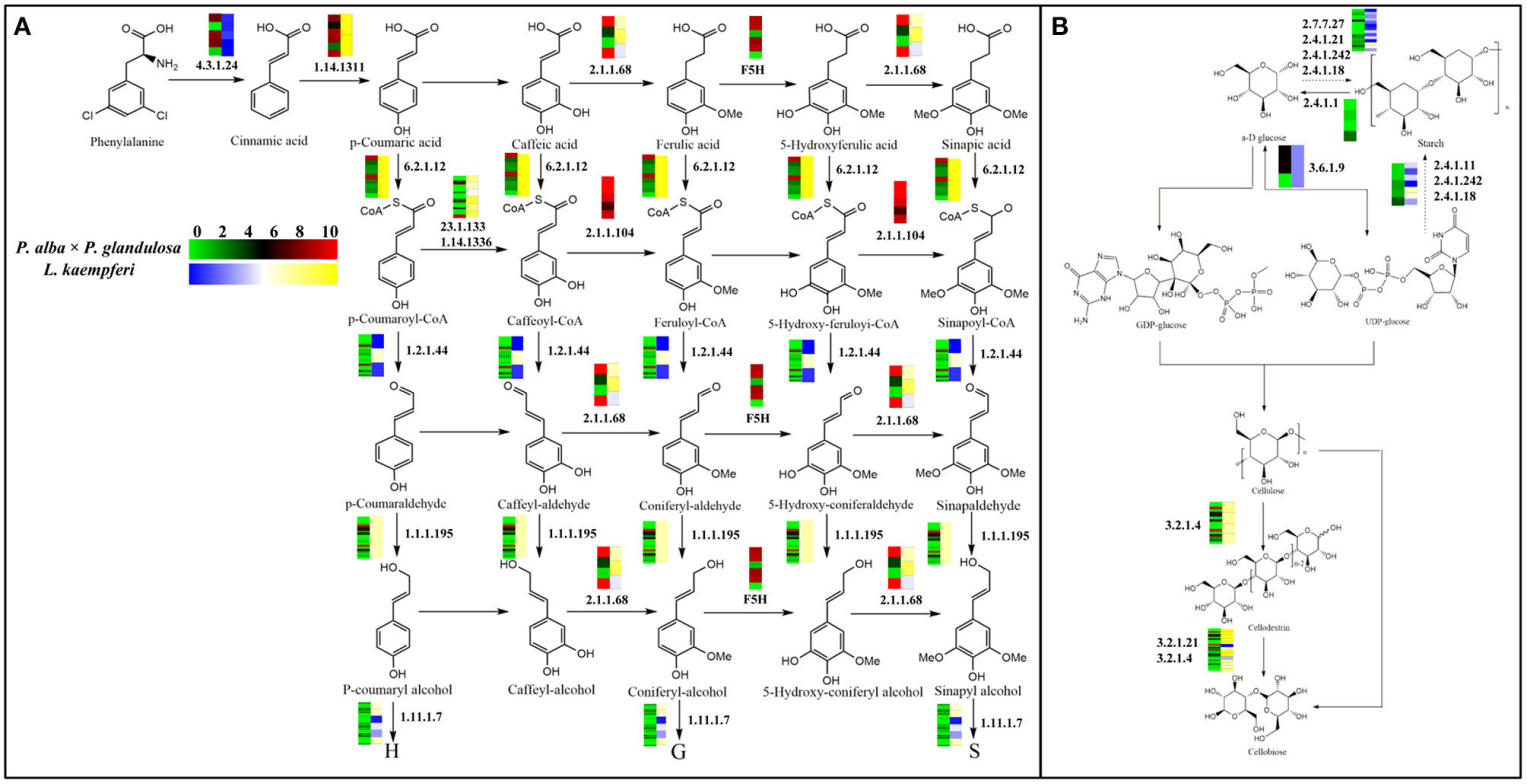
Gene expression model for the lignin and cellulose biosynthesis pathway. **(A)** Branch of phenylpropanoid biosynthesis pathway that is related to lignin biosynthesis. **(B)** Branch of starch and sucrose metabolism pathway that is related to cellulose biosynthesis. The heatmaps in each pathway are visualized according to gene expression levels. In the color bar, red indicates a high expression level and green a low expression level in *P. alba* × *P. glandulosa*; yellow corresponds to high expression level and blue a low expression level in *L. kaempferi*. The dotted arrows in the pathways denote an indirect linkage. The solid arrows refer to a direct relationship during compound synthesis. Numbers in the graph are EC identifier of corresponding enzyme.

Here, we selected two main pathways related to wood formation: phenylpropanoid biosynthesis, and starch and sucrose metabolism (Figure 4), and went on to associate each with those xylem-specifically expressed genes according to the KEGG results. Evidently, in both pathways, most of those genes were active in the same step during lignin and cellulose synthesis processes in the two species. Yet differences did exist between these tree species. For instance, in the phenylpropanoid biosynthesis pathway, during the transformation from ferulic acid to 5-hydroxyl ferulic acid, coniferyl-aldehyde to 5-hydroxy-coniferaldehyde, and coniferyl-alcohol to 5-hydroy-coniferyl alcohol, we found that six highly expressed genes (homologous to *FAH1*) participated in these steps in *P. alba* × *P. glandulosa*. These genes encode enzymes that catalyze the hydroxylation of coniferyl alcohol and coniferaldehyde during syringyl lignin formation (Wu et al., 2018). In stark contrast, such genes were apparently absent in *L. kaempferi* (Figure 4A; Supplementary Tables 4A,B).

During the biosynthesis process of cinnamic acid from phenylalanine, most genes in *P. alba* × *P. glandulosa* were highly expressed, whereas most genes in *L. kaempferi* were expressed at low levels. In the synthesis process converting cafferyl-CoA to feruloyl-CoA, and 5-hydroxy-feruloyl CoA to sinapoyl-CoA, we identified five active genes (homologous to *CCoAOMT1* and *CCOAMT*) in *P. alba* × *P. glandulosa* (Figure 4A; Supplementary Tables 4A,B), known to related to lignin content and composition (Xie et al., 2019). In the starch and sucrose metabolism pathway, four genes (homologous to*PHS1* and *DPE2*) were expressed in *P. alba* × *P. glandulosa*, these participating in the transformation from starch to α-D glucose (Figure 4B; Supplementary Tables 4A,B). These divergent results may explain why the compound synthesis mechanism differs between gymnosperm and angiosperm species during their wood formation.

Although both tree species also harbor homologous genes involved in the same steps, their respective expression patterning was not totally identical, with certain discrepancies among them. For example, during the formation of an alcohol-derived compound from an aldehyde-derived compound, we distinguished 21 genes—homologous to *CAD4, 6, 7, 9, K9L2.15*,0.1*8*,0.1*9*,0.2*0, MEE23, T17H7.1*, and *FOX2*—expressed in these steps in *P. alba* × *P. glandulosa*. Among them, CAD (cinnamyl alcohol dehydrogenase) family members are involved in lignin biosynthesis and catalyze the final step specific for the production of lignin monomers (Kim et al., 2004). A member of the BBE-like family*, T17H7.1*, mediates the oxidation of cinnamyl alcohol and of *p*-hydroxylated derivatives of cinnamyl alcohol during the monolignin metabolism process (Daniel et al., 2015). Yet only Lkgene2978 (homologous to *F19I3.4*) was expressed in *L. kaempferi* (Figure 4A, Supplementary Tables 4A,B). This disparity between species suggests that *P. alba* × *P. glandulosa* relies on a more complicated mechanism than the gymnosperm species during these steps of wood formation. Nevertheless, despite belong to different phyla, *P. alba* × *P. glandulosa* and *L. kaempferi* have expression models with notable similarities. For example, some genes found (homologous to *T16L4.190*) participated in the reciprocal transformation between α-D glucose and UDP-glucose in both *P. alba* × *P. glandulosa* and *L. kaempferi* (Figure 4B; Supplementary Table 4B). This emphasizes that different species within the same phyla could be similar in some aspects.

### WGCNA Analysis and Co-expression Network Building for Wood-Formation Related Genes in the Two Tree Species

To better understand the relationships among those xylem-specifically expressed genes, we performed a WGCNA analysis (Lukens and Downs, 2012) (Supplementary Figure 2). For *P. alba* × *P. glandulosa*, its 13,907 xylem-specifically expressed genes could be divided into 20 dynamic modules. After merging them, eventually we obtained four modules: a dark magenta module, an orange module, a saddle brown module, and a white module (Supplementary Figure 2A). For *L. kaempferi*, its 2,596 xylem-specifically expressed genes were divided into 22 dynamic modules. After merging those, three modules were obtained: a dark green module, a green module, and a midnight blue module (Supplementary Figure 2B).

**Figure 5:**
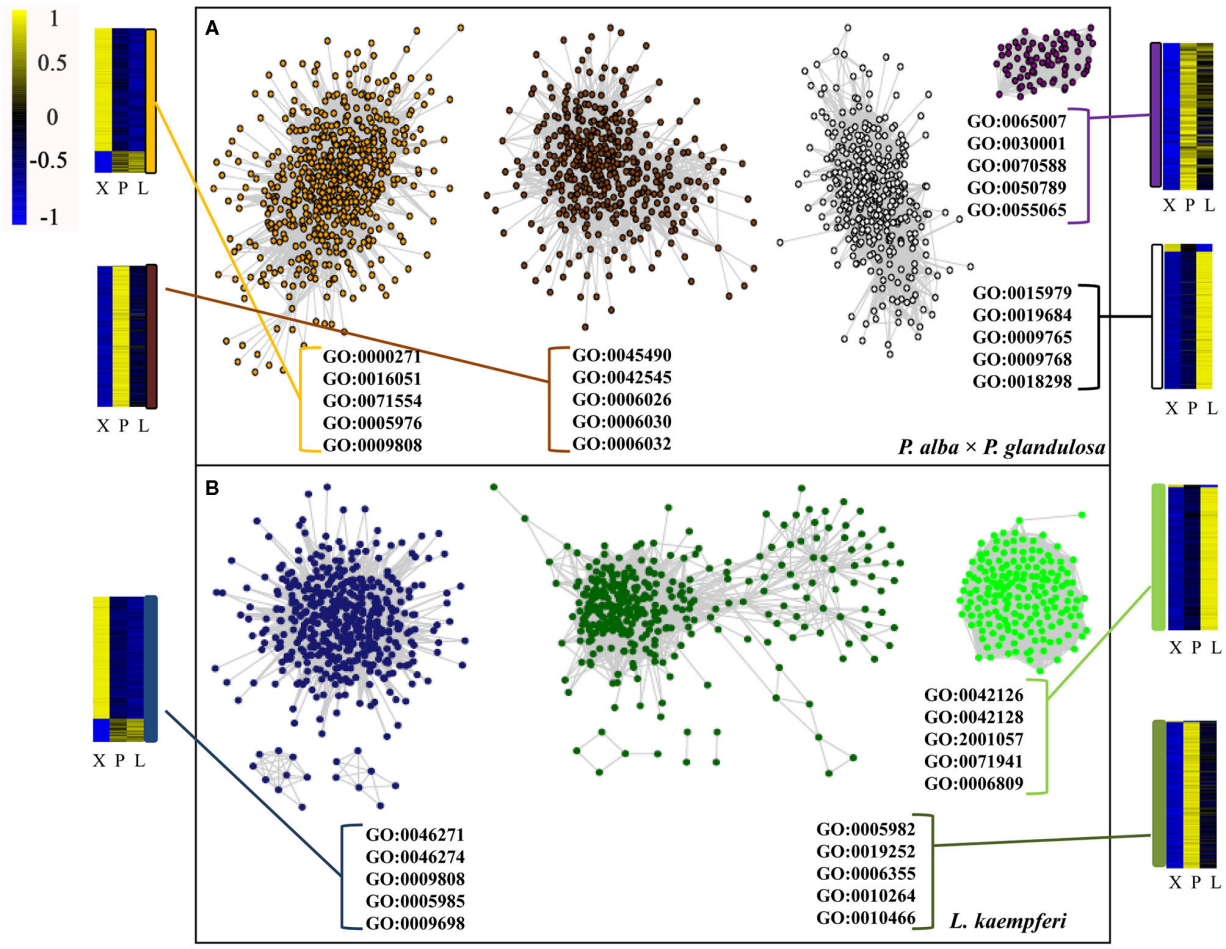
Co-expression network and GO analysis for each module in the two tree species. **(A)** Co-expression network in *P. alba* × *P. glandulosa*. **(B)** Co-expression network in *L. kaempferi*. In these networks, only the top 50 000 gene co-expression pairs are displayed. Only the top five GO terms under the biological process category appear in each module. The heatmaps are for gene expression levels in the different modules.

After doing the WGCNA analysis for each selected module per species, the top 50,000 gene pairs ordered by the edge weight coefficient were chosen to build a gene co-expression network (Figure 5; Supplementary Table 5). A follow-up GO analysis was then performed for each module, from which only the top five GO numbers in BP are shown graphically (Figure 5). For *P. alba* × *P. glandulosa*, the GO analysis showed that more of its wood formation-related genes were clustered in the orange module. Among these top five GO terms, GO: 0009808 had a relationship with lignin biosynthesis (Figure 5; Supplementary Table 5C). In *L. kaempferi*, the genes were significantly enriched in starch metabolic process (GO: 0005982) and starch biosynthetic process (GO: 0019252) in the dark green module (Figure 5; Supplementary Table 5I), as were the phenylpropanoid catabolic process (GO: 0046271), lignin catabolic process (GO: 0046274), lignin metabolic process (GO: 0009808), and phenylpropanoid metabolic process (GO: 0009698) in the midnight blue module (Figure 5; Supplementary Table 5M).

**Figure 6:**
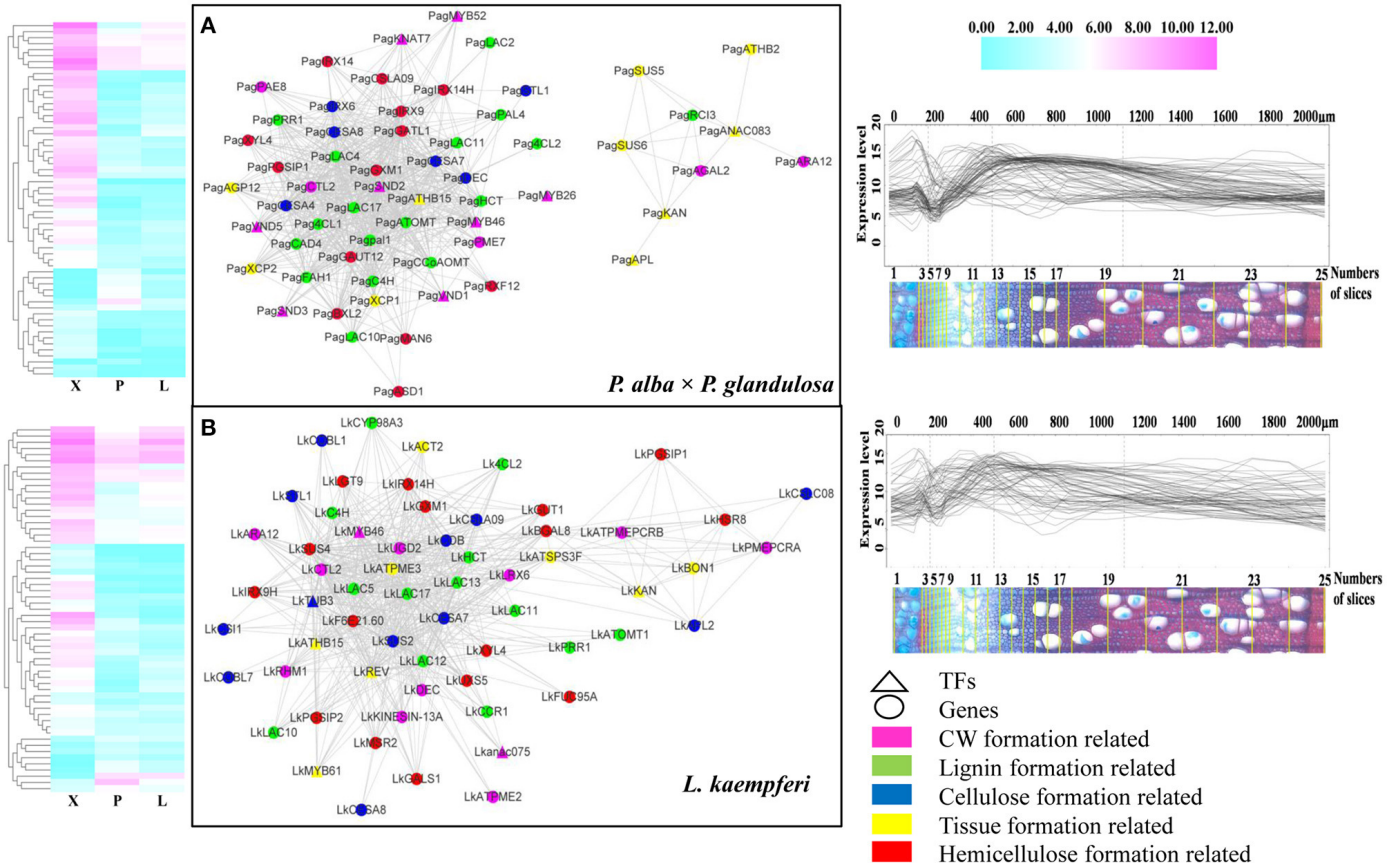
Co-expression network of wood formation-related genes in the two tree species. **(A)** Network for wood formation-related genes in *P. alba* × *P. glandulosa*. **(B)** Network for wood formation-related genes in *L. kaempferi*. The heatmaps (left) and gene expression abundance curves (right) depict the corresponding genes in each co-expression network. The scales above the expression curves indicate the slice position; the scales under expression curves indicate the number of slices. Gene expression abundance curves were derived and drawn using an online tool (http://aspwood.popgenie.org).

Combining the GO and KEGG results and the BLASTn to *Arabidopsis*, we then screened the wood formation-related genes among all xylem-specifically expressed genes and built the former's co-expression network in the two tree species (Figure 6). In the resulting co-expression network, 36 co-expressed genes were specific to *P. alba* × *P. glandulosa* (Supplementary Table 6A), 34 co-expressed genes were specific to *L. kaempferi* (Supplementary Table 6B), and 27 genes were shared by the two species (Supplementary Table 6C). Of the 36 specifically expressed genes in *P. alba* × *P. glandulosa*, four NAC members (*VND1, VND5, SND2, SND3*), two MYB members (*MYB26, MYB52*), two laccase members (*LAC2, LAC4*), and two cysteine protease members (*XCP1, XCP2*) were obtained. Among these, *SND2* is known to play a crucial role in the biosynthesis of cellulose, mannan, and xylan, in addition to cell wall modification and lignin polymerization, but not so in monolignol biosynthesis. The *SND2* promotes the up-regulation of several TF genes, such as *MYB103* and *SND1*, and it occupies a subordinate position in the transcriptional regulatory network (Hussey et al., 2011). *SND3* is directly activated by *SND1/NST1* and *VND6/VND7*, yet there is still no evidence to prove *SND3* is a direct target of *VND6* or *VND7* (Hussey et al., 2011). Both *VND1* and *VND5* are specifically expressed in vessels, where they activate the expression of secondary wall biosynthetic genes for cellulose, xylan, and lignin and concomitantly induce the ectopic deposition of secondary walls (Zhou et al., 2014).

We also obtained two notable cysteine protease gene family members, *XCP1* and *XCP2*, in the co-expression network of *P. alba* × *P. glandulosa*. This gene family is involved in other PCD (programmed cell death) events that contribute to plant organ senescence, plant defense responses, and nutrient mobilization. Both *XCP1* and *XCP2* are frequently used as markers for xylogenesis, and prominently positioned in models of TEs' (tracheary elements) PCD to carry out their autolysis. They are dispersed in the cytoplasmic compartment and imported into the vacuole (Funk et al., 2002; Avci et al., 2008; Zhang et al., 2014). In our results, however, the absence of *XCP1* and *XCP2* in *L. kaempferi* (Supplementary Table 6B) may have led to a TE PCD process unlike that which characterizes *P. alba* × *P. glandulosa*.

In 34 specifically expressed genes in *L. kaempferi* (Supplementary Table 6B), one NAC member (*ANAC075*), three laccase members (*LAC5, LAC12, LAC13*), and one *MYB* member (*MYB61*) were obtained. Among them, we know that *ANAC075* promotes the expression of the secondary wall-associated TF called *MYB46* (Endo et al., 2015) and functions upstream of *NAC030, VND7, NAC101, VND6, LBD30*, and *ASL19* (Sakamoto and Mitsuda, 2015). We then verified the co-expression relation for all the genes forming the network via AspWood web resources (http://aspwood.popgenie.org). We used BLASTn to find the homologous genes in both tree species (Supplementary Tables 6D,E). As Figure 6 shows, for *P. alba* × *P. glandulosa*, 59 out of 63 genes in its network were co-expressed in xylem (Figure 6A; Supplementary Table 6D). Similarly, in *L. kaempferi*, 57 out of 64 genes in its network were co-expressed in xylem (Figure 6B, Supplementary Table 6E). For either tree species, the genes in the co-expression network corresponded to actual co-expression relationships present across xylem tissue (≥ 300 μm). This suggests our co-expression networks were robust and valid for inference.

### Comparison of Alternative Splicing Mode for Part of Wood-formation Related Genes in the Two Tree Species

**Figure 7:**
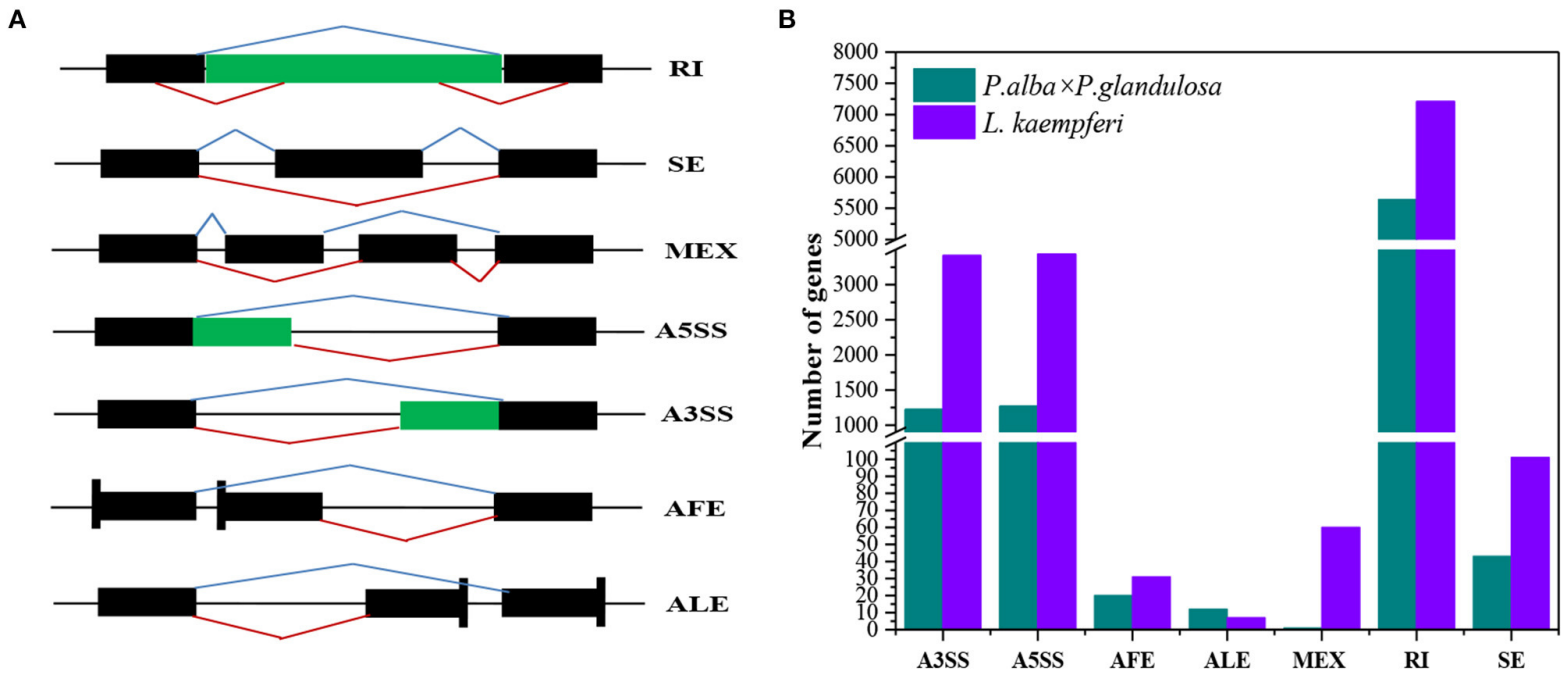
Comparison of different alternative splicing events between the two tree species. **(A)** Splicing patterns of seven kinds of alternative splicing. **(B)** Distributions of seven kinds of alternative splicing in each of the two tree species. A3SS, alternative 3′ splice site; A5SS, alternative 5′ splice site; AFE, alternative first exon; ALE, alternative last exon; MEX, mutually exclusive exon; RI, retained intron; SE, skipped exon. Black blocks are exons, while the green blocks and black lines represent introns. Blue broken lines above the block(s) represent normal transcripts; red broken lines are the alternative splicing events.

**Figure 8:**
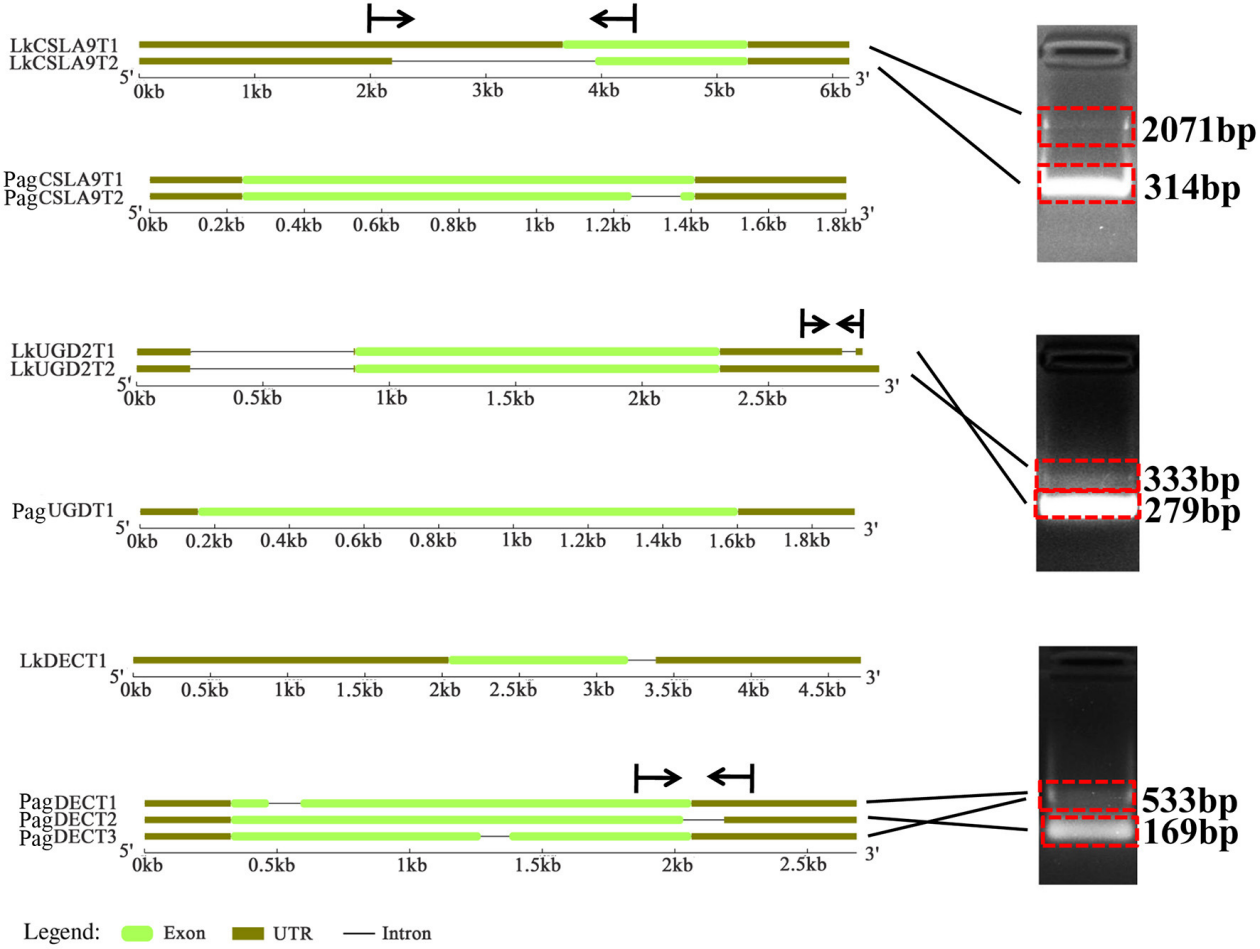
Comparison of the gene structure for three common genes and their variants in the two tree species. Vertical lines and arrows indicate the position of designed primers and amplification direction of fragments, respectively.

Seven AS forms were detected, namely the alternative 3′ splice site (A3SS), alternative 5′ splice site (A5SS), alternative first exon, alternative last exon, mutually exclusive exon, retained intron (RI), and skipped exon (Figure 7A). We derived an AS model of genes expressed in xylem of *P. alba* × *P. glandulosa* and *L. Kaempferi* (Figure 7B). This showed similar proportions for different tree species, in that A3SS, A5SS, and RI are the main splicing models. Further investigation of the common wood formation-related genes for both species showed that *CSLA9, UGD2*, and *DEC* featured AS. The verification of AS in both tree species was pursued and these results confirmed that *CSLA9* in *L. kaempferi* accorded with the putative AS results presented in Figure 8. Although our bioinformatic analysis indicated the gene *CSLA9* also can undergo AS in *P. alba* × *P. glandulosa*, our RT-PCR result failed to detect it. We also confirmed AS variants of *UGD2* in *L. kaempferi*, and those of *DEC* in *P. alba* × *P. glandulosa* (Figure 8). Collectively, these results demonstrated there is differential post-transcriptional regulation in *P. alba* × *P. glandulosa* and *L. kaempferi*, which could point to the possible causes for the differing wood structure in these two kinds of trees.

## Discussion

In comparison to gymnosperm species, angiosperms underwent massive adaptive radiation to supplant the gymnosperms as the world's dominant vascular plant group (Sanderson and Donoghue, 1994; Pavy et al., 2012). During evolutionary process, angiosperm wood form their own structure characters which is mostly composed of vessels, rays, fibers, and parenchyma cells and sharply contrast to the relatively simple gymnosperms' wood which mainly consists of tracheid and rays (Zhang et al., 2017). Besides these wood structural differences, gymnosperms and angiosperms also differ in their HD-Zip III genes and hemicelluloses ingredients (Côté et al., 2010); in the binding of multiple xylan chains to adjacent planes of the cellulose fibril (Marta et al., 2016); metabolically, in the formation of syringyl lignin (Nakamura et al., 1974), and; in their cell wall-associated peroxidases involved in xylem lignification (Mcdougall, 2001).

Gene co-expression represents interactive relationships among genes to a certain extent (Mitsuda and Ohme-Takagi, 2009). For TFs, their co-expression is indicative of coding function-related or protein–protein interactions (Mitsuda and Ohme-Takagi, 2009), and more generally, co-expressed genes may have up- or down-stream relationships in the transcriptional cascade (Hirai et al., 2007). In the constructed co-expression network, we detected similar and different co-expressed genes between the two tree species (Supplementary Tables 6A–C). Although both species do share some common genes, these genes still had some differences in their structure. For example, in their investigation of the structure of HD-Zip III family between angiosperms and gymnosperms, Côté et al. (2010) found that gymnosperm sequences derived from lineages that diverged earlier than angiosperm sequences, and that some sequences were lost in angiosperms indicates the full-length cDNA is longer in gymnosperms than angiosperms. Consistent with the above research (Côté et al., 2010), our gene structure analysis showed that *UGD2, CSLA9*, and *DEC* in *L. kaempferi* are longer than in *P. alba* × *P. glandulosa*, especially in the UTR region, suggesting lost sequences in *P. alba* × *P. glandulosa*.

Both FAH1 and CYP98A3 are members of P450 (Cytochrome P450). We found that *FAH1*, also named *F5H*, was expressed specifically in the xylem of *P. alba* × *P. glandulosa* (Figure 4A, Supplementary Table 6A). In contrast, *CYP98A3* was expressed only in the xylem of *L. kaempferi* (Supplementary Table 6B) where it could catalyze the hydroxylation of shikimic acid and quinic acid to form monolignin. Its substrate mainly includes *p*-coumarate, *p*-coumaraldehyde, and *p-*coumaroyl methyl ester, which differs from *FAH1*. Other studies also found that *CYP98A3* could participate in the biosynthesis process of the coumarins scopoletin and scopolin (Schoch et al., 2001; Abdulrazzak et al., 2006). Previous research indicated that *FAH1* is associated with S-lignin monomer formation, in that the expression of *FAH1* could increase the ratio of S/G (Boerjan et al., 2003). In our study, *FAH1* was only present in *P. alba* × *P. glandulosa*, indicating a high syringyl lignin content and high proportion of S/G in this species. This result is consistent with work by Boerjan et al. (2003), which revealed that most dicotyledonous angiosperms (hardwood) are rich in G and S lignin monomers yet poor in the H lignin monomer. In gymnosperms (softwood), the G lignin monomer is the main lignin monomer and S and H lignin monomers' content is low (Boerjan et al., 2003). This implies a pronounced difference in the lignin monomer formation process between angiosperm and gymnosperm plants. We found *SND2* was specific to *P. alba* × *P. glandulosa* whereas *ANAC075* was specific to *L. kaempferi*. Both *SND2* and *ANAC075* can influence glucose and xylose and lignin contents, but *ANAC075* has a greater transcriptional activation ability than does *SND2*, suggesting a different wood formation mechanism between angiosperm and gymnosperm species (Sakamoto and Mitsuda, 2015).

Although MYB family members were found in the studied two tree species, *MYB26* and *MYB52* were specifically expressed in the xylem of *P. alba* × *P. glandulosa* (Supplementary Table 6A). *MYB26* localizes to the nucleus and regulates endothelial development and the secondary wall- thickening process (Yang et al., 2007). *MYB52* is involved in both the ABA response and cell wall biosynthesis, and its overexpression in *Arabidopsis* improves this plant's drought tolerance and salt-sensitivity (Park et al., 2011). Even so, *MYB52, SND2*, and *SND3* together with *KAN7* can also influence the secondary cell wall-thickening process in fiber cells under the control of *SND1* (Zhong et al., 2008). *MYB61* mainly participates in xylem formation, by inducing qualitative changes to the xylem cell structure and lateral root development (Romano et al., 2012). The characteristic expression model in *P. alba* × *P. glandulosa* illustrated a distinctive wood formation process when compared with that of gymnosperm species. Laccase genes were reportedly active in the late stage of lignin formation and could promote lignification of the secondary xylem cell walls (Brown et al., 2005) and their different subcellular localization implies different functions among the laccase members. For instance, some research indicates that laccase can play additional roles in plants that go beyond the lignification process (Cai et al., 2006). In our results, we found co-expression of laccase members *LAC2* and *LAC4* in *P. alba* × *P. glandulosa*, and likewise that of *LAC5, LAC12*, and *LAC13* in *L. kaempferi*, which suggests laccase genes may be the key factors influencing wood structure. Fine-scale distinctions among these laccase genes' functioning deserve further investigation.

Alternative splicing (AS) is a criticalpost-regulation process for the expression of genes. In this way, different transcripts could encode different proteins to perform various functions and this contributes to the variety of transcripts and proteins available for use (Chen and Manley, 2009; Reddy and Yamile, 2013). Researchers have since discovered that approximately 30 to 60% of mRNAs have variants (Zhang et al., 2010; Shen et al., 2014). According to our results, AS events characterized about 14.5% of the genes (8,207 of 56,711) in *P. alba* × *P. glandulosa*, but this was threefold greater, at 46.7% (14,259 of 30,726 genes), in *L. kaempferi*. Compared with other research (Zhang et al., 2010; Shen et al., 2014), our results uncovered a lower proportion of AS events in *P. alba* × *P. glandulosa*, perhaps due to the quality of its references. A previous study also showed that the retained intron is the main AS form, constituting approximately 60% of all AS events in plants (Syed et al., 2012). Similarly, in our study, the chief form of AS encountered was the retained intron, occurring in about 68.7% of genes (5,639 out of 8,207) in *P. alba* × *P. glandulosa* and 50.6% of those (7,209 out of 14,259) in *L. kaempferi*.

## Conclusion

In this study, we compared the xylem-specifically expressed genes between *P. alba* × *P. glandulosa* and *L. kaempferi* trees on the transcriptional level and post-transcriptional level. Our results showed that differences exist in the enriched GO terms and KEGG pathways, gene expression models of the lignin and cellulose biosynthesis-related pathway, co-expression relationships, and alternative splicing forms, which together indicates different wood formation processes between *P. alba* × *P. glandulosa* and *L. kaempferi*. Our research provides a timely foundation for the further discovery and elucidation of differing wood formation mechanisms between angiosperm and gymnosperm species.

## Data Availability Statement

The original contributions presented in the study are publicly available. This data can be found at: NCBI repository, accession number: PRJNA723483.

## Author Contributions

HL and XD conceived and designed the study. QW and XD collected the plant materials. XD, QW, and HL extracted the RNA. HP, XD, and HL conducted the validation of RNA-Seq and alternative splicing events. HL and GC performed the bioinformatics analyses and carried out the data visualization. HL and XD wrote the manuscript. All of the authors revised and approved the manuscript.

## Conflict of Interest

The authors declare that the research was conducted in the absence of any commercial or financial relationships that could be construed as a potential conflict of interest.
