# Supplementary material for: Investigation Into Different Wood Formation Mechanisms Between Angiosperm and Gymnosperm Tree Species at the Transcriptional and Post-transcriptional Level
Source: Front Plant Sci. 2021 Jul 2;12:698602. doi: 10.3389/fpls.2021.698602 (PMC8283789; doi:10.3389/fpls.2021.698602)
Supplement: Supplementary file 2 [file Data_Sheet_2.docx]

Supplementary Material

# Supplementary Data

# Supplementary Figures and Tables

For more information on Supplementary Material and for details on the different file types accepted, please see [here](http://home.frontiersin.org/about/author-guidelines#SupplementaryMaterial). Figures, tables, and images will be published under a Creative Commons CC-BY licence and permission must be obtained for use of copyrighted material from other sources (including re-published/adapted/modified/partial figures and images from the internet). It is the responsibility of the authors to acquire the licenses, to follow any citation instructions requested by third-party rights holders, and cover any supplementary charges.

## Supplementary Figures

**
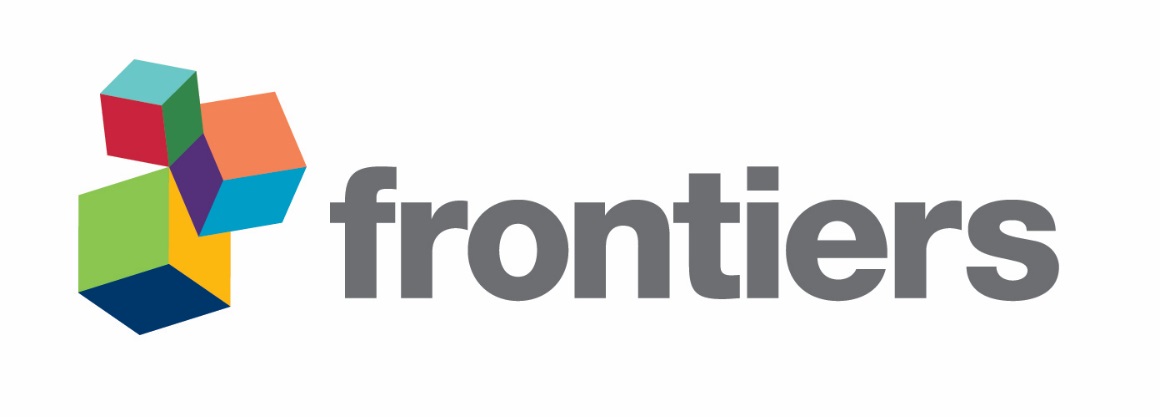
**


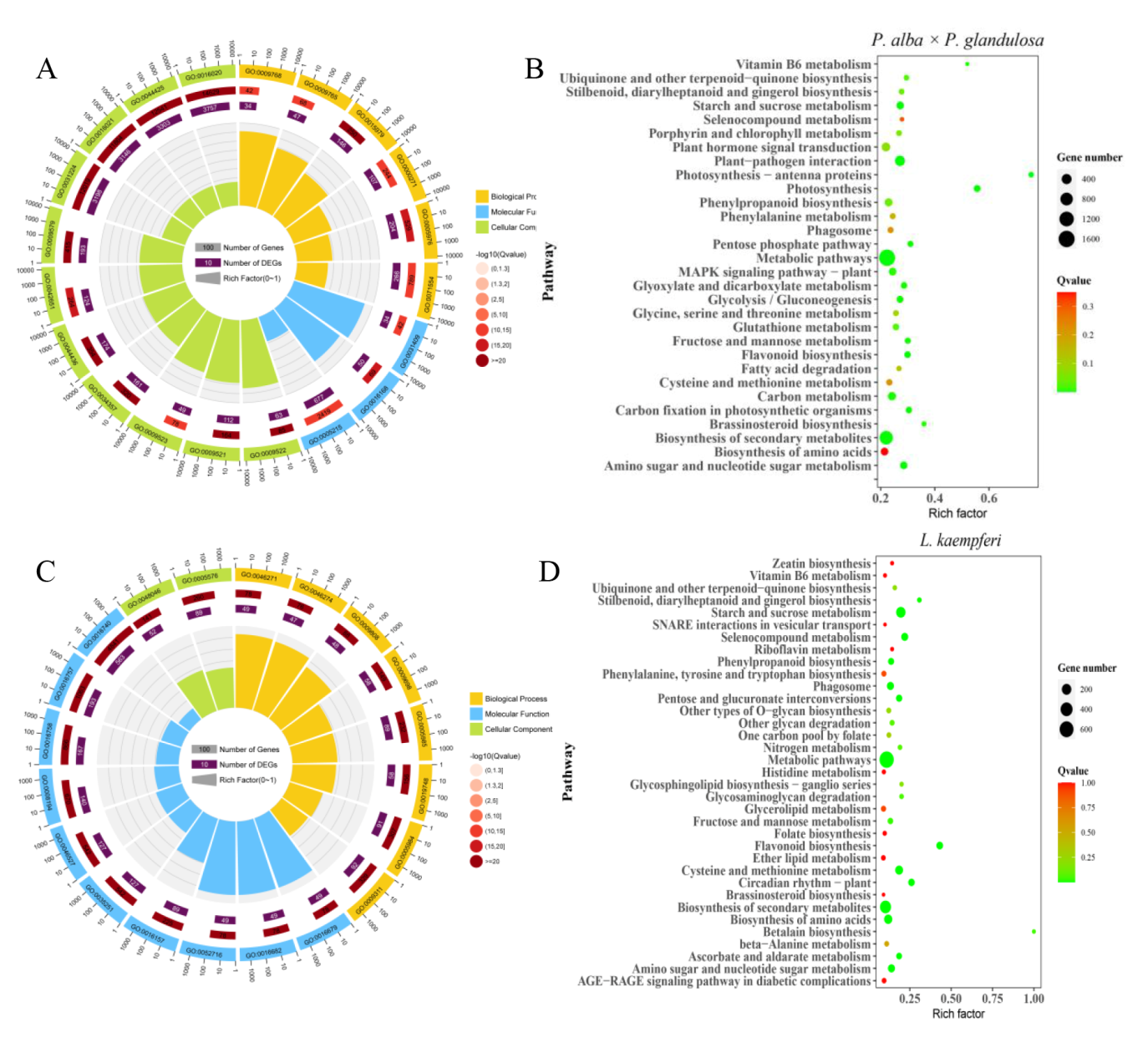


**Supplementary Figure 1.** GO and KEGG analysis for two tree species. A and C display a more detailed classification of GO analysis for *P. alba × P. glandulosa* and *L. kaempferi* respectively. B and D showed KEGG results for *P. alba × P. glandulosa* and *L. kaempferi* respectively. Topological structure of top 3 highest enrichment factor GO terms (molecular function, cellular component and biological process) were showed in A and C. Top30 pathways were showed in the graph B and D. The dot size in the graph represented the gene numbers in each pathway. Rich factor and *Q* value represented the extent of gene enriched in the KEGG database. Both *Q* value in GO and KEGG analysis were standardized by –log_10_ (*Q* value) algorithm.


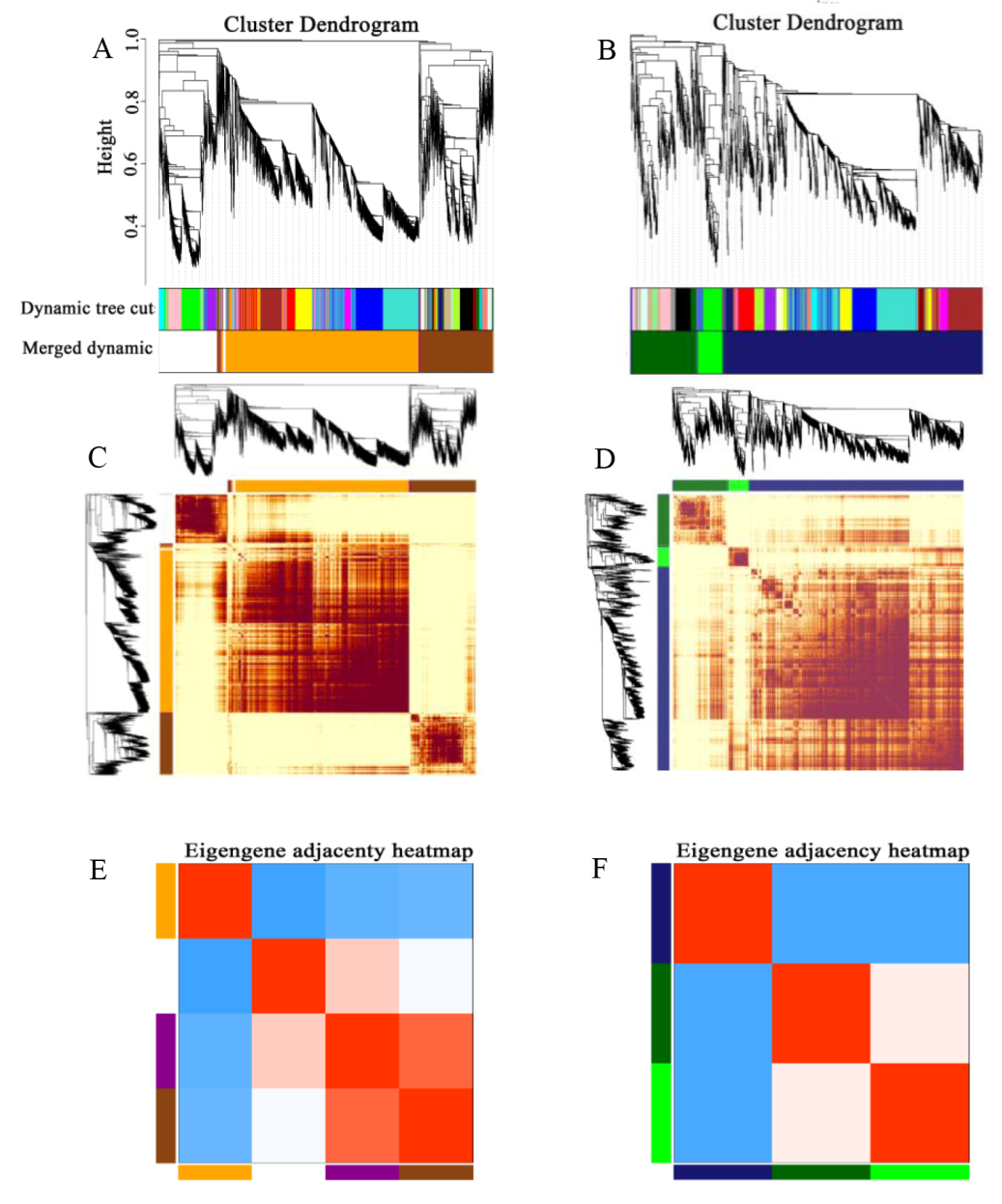


**Supplementary Figure 2.** Result of WGCNA analysis for two tree species. A, C and E are WGCNA analysis results of *P. alba × P. glandulosa*. B, D and F are WGCNA analysis results of *L. kaempferi*. The different colors in A and B represent different modules. C and D are heatmaps of gene co-expression network. The heatmaps represent adjacencies among genes in analysis: one single gene corresponds to other genes in each row and column. Dark color means high correlation between two genes. Light-luminance color means low correlation between two genes. E and F are correlation analyses result of different module for each species. The red color means high correlation between two modules. The blue color means low correlation between two modules.

## Supplementary table

**Supplementary Table S1.** Primers used in validation of RNA-seq and alternative splicing events. **Supplementary Table S1A.** Primers for validation of RNA-seq; **Supplementary Table S1B.** Primers for verification of alternative splicing.

**Supplementary Table S2.** Differentially Expressed Gene analysis in two tree speices. **Supplementary Table S2A.** Differentially Expressed Gene analysis result in XvsP group of *P. alba × P. glandulosa*; **Supplementary Table S2B.** Differentially Expressed Gene analysis result in XvsL group of *P. alba × P. glandulosa*; **Supplementary Table S2C.** Differentially Expressed Gene analysis result in XvsP group of *L. kaempferi*; **Supplementary Table S2D.** Differentially Expressed Gene analysis result in XvsL group of *L. kaempferi*.

**Supplementary Table S3.** GO and KEGG analysis results of two tree species. **Supplementary Table S3A.** GO analysis for up and down regulated genes in xylem of *P. alba × P. glandulosa*; **Supplementary Table S3B.** KEGG analysis for up and down regulated genes in xylem of *P. alba × P. glandulosa*; **Supplementary Table S3C.** GO analysis for up and down regulated genes in xylem of *L. kaempferi*; **Supplementary Table S3D.** KEGG analysis for up and down regulated genes in xylem of *L. kaempferi.*

**Supplementary Table S4.** Gene distribution in wood-formation related pathway in two tree speices. **Supplementary Table S4A.** Gene distibution in wood formation related pathway in *P. alba × P. glandulosa*; **Supplementary Table S4B.** Gene distibution in wood formation related pathway in *L. kaempferi.*

**Supplementary Table S5.** GO and KEGG analysis results in different module of two tree species. **Supplementary Table S5A.** GO analysis result in darkmagenta moduel of *P. alba × P. glandulosa*; **Supplementary Table S5B.** KEGG analysis result in darkmagenta moduel of *P. alba × P. glandulosa*; **Supplementary Table S5C.** GO analysis result in orange moduel of *P. alba × P. glandulosa*; **Supplementary Table S5D.** KEGG analysis result in orange moduel of *P. alba × P. glandulosa*; **Supplementary Table S5E.** GO analysis result in saddlebrown moduel of *P. alba × P. glandulosa*; **Supplementary Table S5F.** KEGG analysis result of saddlebrown moduel of *P. alba × P. glandulosa*; **Supplementary Table S5G.** GO analysis result in white moduel of *P. alba × P. glandulosa*; **Supplementary Table S5H.** KEGG analysis result in white moduel of  *P. alba × P. glandulosa*; **Supplementary Table S5I.** GO analysis result in darkgreen moduel of *L. kaempferi*; **Supplementary Table S5J.** KEGG analysis result in darkgree moduel of *L. kaempferi*; **Supplementary Table S5K.** GO analysis result in green moduel of *L. kaempferi*; **Supplementary Table S5L.** KEGG analysis result in green moduel of *L. kaempferi*; **Supplementary Table S5M.** GO analysis result in midnightblue moduel of *L. kaempferi*; **Supplementary Table S5N.** KEGG analysis result in midnightblue moduel of *L. kaempferi*.

**Supplementary Table S6.** Comparison of wood-formation related genes co-expression network of two tree species. **Supplementary Table S6A.** Wood formation related genes in co-expression network of *P. alba × P. glandulosa*; **Supplementary Table S6B.** Wood formation related genes in co-expression network of *L. kaempferi*; **Supplementary Table S6C.** Specifically expressed genes in co-expression network of *P. alba × P. glandulosa*; **Supplementary Table S6D.** Specifically expressed genes in co-expression network of *L. kaempferi*; **Supplementary Table S6E.** Common genes in co-expression network of *P. alba × P. glandulosa* and *L. kaempferi*.
