# Supplementary figures and images for: Investigation Into Different Wood Formation Mechanisms Between Angiosperm and Gymnosperm Tree Species at the Transcriptional and Post-transcriptional Level
Source: Front Plant Sci. 2021 Jul 2;12:698602. doi: 10.3389/fpls.2021.698602 (PMC8283789; doi:10.3389/fpls.2021.698602)

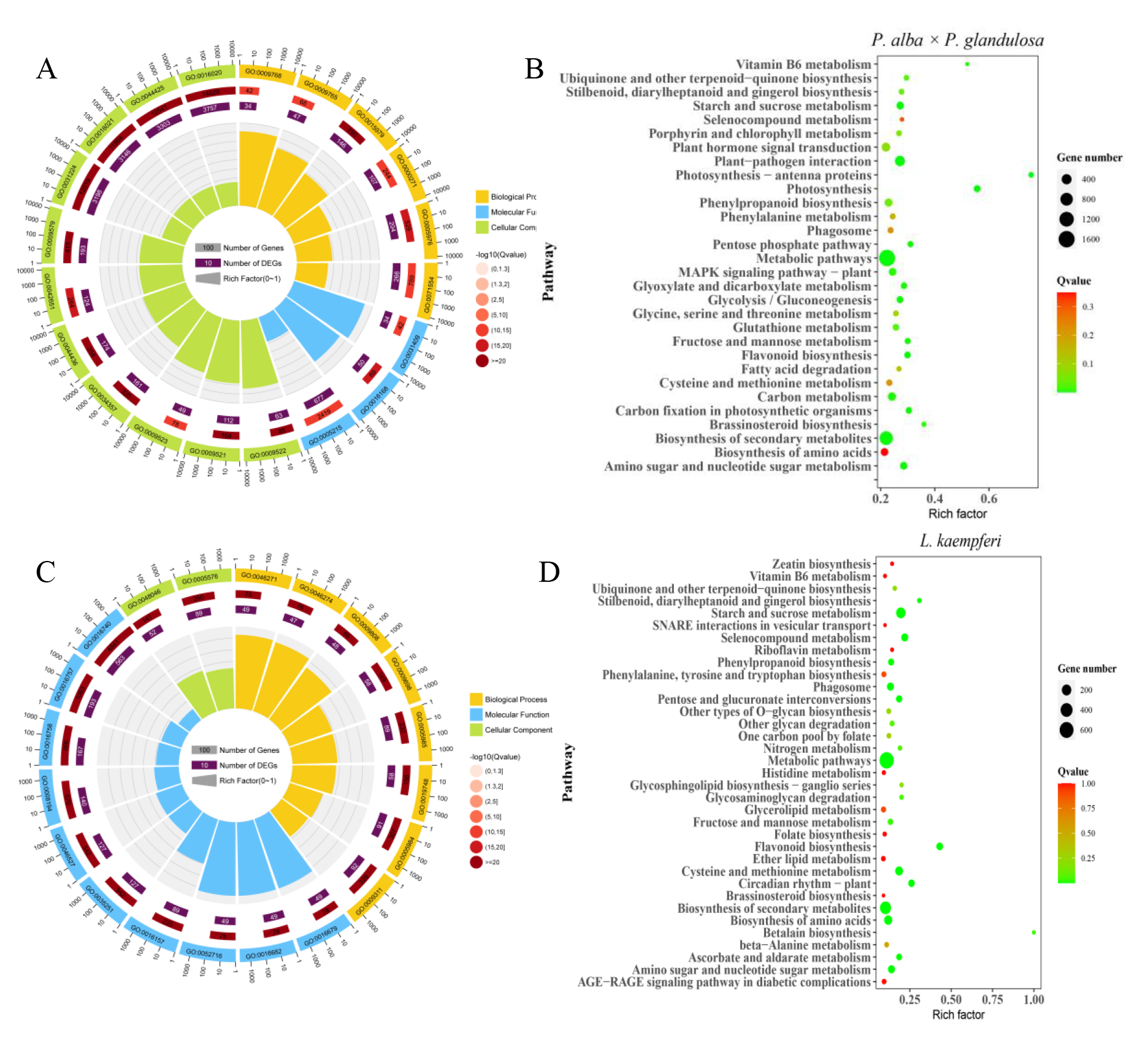

Supplement: Supplementary file 3 [file Image_1.TIF]

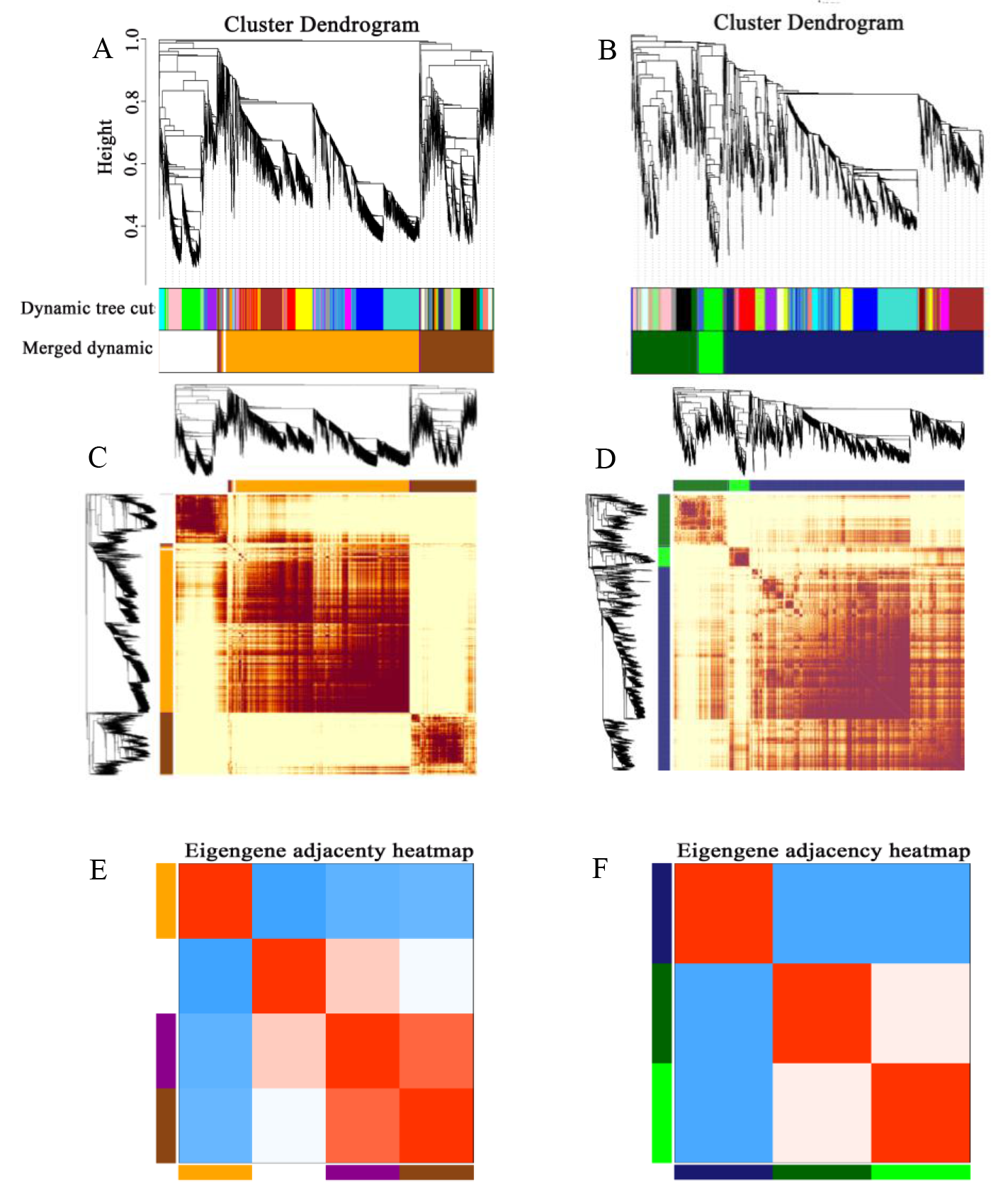

Supplement: Supplementary file 4 [file Image_2.TIF]
